# The effect of subjective and objective social class on health-related quality of life: new paradigm using longitudinal analysis

**DOI:** 10.1186/s12955-015-0319-0

**Published:** 2015-08-08

**Authors:** Young Choi, Jae-Hyun Kim, Eun-Cheol Park

**Affiliations:** Department of Public Health, Graduate School, Yonsei University, 50 Yonsei-ro, Seodaemun-gu, Seoul, 120-752 South Korea; Department of Preventive Medicine, College of Medicine, Yonsei University, 50 Yonsei-ro, Seodaemun-gu, Seoul, 120-752 South Korea; Institute of Health Services Research, College of Medicine, Yonsei University, 50 Yonsei-ro, Seodaemun-gu, Seoul, 120-752 South Korea

## Abstract

**Background:**

To investigate the impact of the gap between subjective and objective social status on health-related quality of life.

**Methods:**

We analyzed data from 12,350 participants aged ≥18 years in the Korean Health Panel Survey. Health-related quality of life was measured by EuroQol-Visual analogue scale. Objective (income and education) and subjective social class (measured by MacArthur scale) was classified into three groups (High, Middle, Low). In terms of a gap between objective and subjective social class, social class was grouped into nine categories ranging from High–High to Low–Low. A linear mixed model was used to investigate the association between the combined social class and health-related quality of life.

**Results:**

The impact of the gap between objective and subjective status on Health-related quality of life varied according to the type of gap. Namely, at any given subjective social class, an individual’s quality of life declined with a decrease in the objective social class. At any given objective social class (e.g., HH, HM, HL; in terms of both education and income), an individual’s quality of life declined with a one-level decrease in subjective social class.

**Conclusion:**

Our results suggest that studies of the relationship between social class and health outcomes may consider the multidimensional nature of social status.

**Electronic supplementary material:**

The online version of this article (doi:10.1186/s12955-015-0319-0) contains supplementary material, which is available to authorized users.

## Introduction

Several studies of health inequality have shown that the traditional measure of socioeconomic status (i.e., education, income, and occupation) is an important predictor of health [1–3]. In general, the lower the SES, the worse the health outcome [4–8]. In addition to the conventional measures of socioeconomic status (SES), subjective social class, which refers to “the individual’s perception of his own position in the social hierarchy,” is a novel indicator of social level [9] and a better predictor of health [10, 11], such as physical [12, 13], mental health [10, 14–16], diseases [17, 18], and mortality [19]. Both social classes affect health-related quality of life or self-reported health. For instance, low educational status or lack of material belongings has been associated with a decrease in health-related quality of life [20]. Similar studies found that low subjective social status was associated with poor self-rated health [21, 22].

In attempt to understand the possible factors linking SES and health, researchers have summarized variables: socio-demographic, economic, environmental, behavioral psychological, and physiological [23]. SES influence health outcomes through its association with behavioral and psychological risk factors. For example, people with low SES experience more depression and poor health behavior than their higher SES counterparts [24]. Moreover, a study show demographic characteristics are also important predictors of health-related quality of life [25].

Health-related quality of life (Health-related quality of life), an individual’s perception of his or her physical, emotional, psychological, and social health, has been broadly used to assess health outcome variables [26]. Indicators of perceived general health were good predictors of an individual’s future health care use and mortality despite the subjective nature of the concept [27, 28]. Indeed, health-related quality of life is an important predictor of mortality [29, 30], morbidity [31, 32], and poor health [33].

The association between social class and health or health-related quality of life is well established. Previous studies have shown that even though education and income are correlated with subjective social level [11, 34–37] and subjective social status is a better predictor of health outcome after traditional measures of SES and other factors [35] are controlled. No studies, however, have investigated how much there is a gap between objective and perceived social status on health outcomes.

In this context, we focused on the multidimensional nature of social class by using a novel approach to measure differences between subjective and objective social status in terms of a disparity, or a gap, between subjective and objective social status. We investigated the association between the gap and health-related quality of life in the Korean population. The investigation of health-related quality of life may be of particular importance in South Korea, where self-rated health is among the lowest in the world despite the fact that overall health in Korea is better than the Organization for Economic Co-operation and Development average [38]. Our findings may provide further understanding of the multidimensional nature of social status in relation to health outcomes.

## Methods

### Study sample

We used data came from the Korean Health Panel Survey (KHPS). KHPS is designed to create nationally representative longitudinal data, which collected between October 2008 and December 2011. Detailed data and information on families and individuals are as the following: demographic and socio-economic characteristics of individuals, health behaviors and health awareness, and health care utilization and expenditure. The panel in the 1st wave for 2008 consisted of 21,283 participants in 6171 households. The numbers of individuals and households in 2009, 2010, and 2011 were 19,154 and 6314; 17,878 and 5956; and 17,037 and 5741; respectively.

For this study, we chose to use the 2009 and 2011 data because it included subjective questions about social class targeting adults aged 18 or above. Among 19,154 participants in 2009, we excluded 6552, 57, and 7 respondents without information on subjective social class, household income, and health-related quality of life, respectively. From 12,538 individuals, 188 individuals without information on health risk and behavior (smoking, physical activity, and chronic disease) were excluded. Thus, our analysis included 12,350 individuals from the 2009 survey. Among 17,037 participants in 2011, we excluded 5274 individuals without information on subjective social class and 5 individuals without information on household income. Thus, the 2011 data included a total of 11,758 individuals.

Nine thousand nine hundred forty-five participants responded on both panel surveys in 2009 and 2011, in addition, 2410 participated in 2009 only and 1819 solely in 2011. Thus, a total number of enrolled subjects in this study were 14,172. A repeated-measurement using linear mixed model was performed for this analysis. Therefore, those who participated once (2009 or 2011) were measured once, and those who participated in both surveys were counted twice.

### Study variables

#### Health-related quality of life (HRQoL)

Many reliable and valid instruments to calculate HRQoL are available. The selection of the most appropriate instrument depends on the population, the outcomes of interest, the purpose of assessment, and the characteristics of the instruments. There are two types of instruments in measuring HRQoL. (1) Generic instruments offer the opportunity to compare results across patient and population cohorts, and some can provide normative or benchmark data from which to interpret results. Widely used generic health status measures are the Short-Form Health Survey (SF-36) [39], the Nottingham Health Profile (NHP) [40], and the five dimensions of the EuroQoL questionnaire (EQ-5D) [41]. (2) Targeted instruments ask questions that focus on the specific condition or treatment under study and, as a result, tend to be more responsive to clinically important changes than generic instruments. Examples of such measures include the Functional Assessment of Cancer Therapy–Lung (FACT-L) developed for use with lung cancer patients [42], the Arthritis Impact Measurement Scale (AIMS) [43], and the Spinal Cord Injury Quality of Life Questionnaire (SCI-QL 23) [44].

In this study, the Euroqol visual analogue scale (EQ-VAS), which is widely used as a strong predictor of global self-rating health status, was used to assess individuals’ health state as an outcome variable. The EQ-VAS records the respondent’s current health on a vertical, visual analogue scale with the endpoints “worst imaginable health state” and “best imaginable health state,” ranging from 0 (worst health state) to 100 (best health state) [45]. This instrument provides a quantitative measure of health outcome as judged by the individual respondent.

#### Objective social class

We focused on two dimensions of objective social class (household income and education level). Equivalized household income is an indicator of the economic resources available to each member of a household. Mean equivalized household income is calculated by adding the equivalized household incomes of all members of a household and dividing by the number of household members, which ensures that the contribution of an individual in a large household is the same as that of a person living alone. Thus, equivalized household income is the total household income adjusted by an equivalence scale to facilitate comparisons between households of different sizes and compositions. The number reflects the fact that a larger household requires a higher level of income than a smaller household to achieve the same standard of living. We calculated household income by dividing the yearly household income by the square root of the number of household members [46]. Household incomes were ranked from lowest to highest using the Statistical Analysis System (SAS) Rank function and grouped into three categories (High, Medium, and Low).

Education level was divided into three categories taking into account the cultural environment of South Korea: middle school or lower (Low), high school (Medium), and college or higher (High).

#### Subjective social class

Subjective social class was measured by asking the respondents to assess their perceived social position using a pictorial representation of a ladder [47]. Pictures of ladders with 10 rungs were shown along with the following instructions: “Think of this ladder as representing where people stand in South Korea. At the top of the ladder are the people who are the best off—those who have the most money, the most education, and the most respected jobs. At the bottom are the people who are the worst off—those who have the least money, least education, and the least respected jobs or no jobs.” The respondents were asked to consider their current situation and rank themselves within the South Korean population. The items were coded so that higher scores indicated higher subjective social class. We ranked self-reported social class from lowest to highest using the SAS Rank function (i.e., High, Medium, and Low).

#### The gap between objective and subjective social status

The gap represents the difference between objective (household income and education level) and subjective social status. We classified the reported gaps into nine categories ranging from high SES (household income or education level) and high subjective social class to low SES and low subjective social class (i.e., High–High, High–Medium, High–Low, Medium–High, Medium–Medium, Medium–Low, Low–High, Low–Medium, and Low–Low).

#### Covariates

Residence was categorized as urban (Seoul, Daejeon, Daegu, Busan, Incheon, Kwangju, or Ulsan) or rural (areas not classified as a city). Employment status was categorized as employed or unemployed, which included housewives and students. Individuals were classified as currently married or never married, with the latter group including respondents who had previously been married or were widowed or divorced. Self-reported depressive symptoms were determined from the response to the question “Have you ever felt sadness or despair that interfered with everyday life for 2 or more continuous weeks during a 1-year time period?” The presence of depressive symptoms was categorized as yes or no. Furthermore, the presence of chronic disease was included in our models, and alcohol use, smoking status, and days of exercise per week were included as covariates. These variables are the level-2 covariates (between-subject).

#### Statistical analysis

Chi-square tests, *t*-test and a longitudinal data analysis were used to investigate the impact of the gap between SES and subjective social class on health-related quality of life. We used a linear mixed model to analyze two waves data nested within individuals. The linear mixed model is a tool for analyzing longitudinal data that arise in areas as diverse as clinical trials, epidemiology, agriculture, economics, and geophysics. This model is explained by the flexibility they offer in modeling the within-subject correlation often present in longitudinal data, and by the handling of both balanced and unbalanced data (i.e., data sets with different numbers of observations per subject, or subjects measured at different time points) [48]. All statistical analyses were conducted using SAS 9.2 (SAS Institute, Inc., Cary, NC, USA), and two-tailed *p* values ≤ 0.05 were deemed statistically significant.

## Results

The baseline (2009) socio-demographic characteristics of the study participants (*n* = 12,350) are shown in Table 1. We found little difference between the distributions of the weighted and unweighted percentages. The mean population EQ-VAS scores were 71.99 (unweighted) and 72.81 (weighted).

**Table 1:** General characteristics of the respondents at baseline (2009)

	Total	Unweighted %	Weighted %	Health-related quality of life (EQ-VAS)			*p* value
				Unweighted mean	Weighted mean	SD	
Sex							<0.0001
Male	5,390	43.6	44.5	74.36	75.10	15.46	
Female	6,960	56.4	55.5	70.16	70.98	16.54	
Age							<0.0001
≤ 29	1,547	12.5	16.5	76.72	76.75	17.17	
30–39	2,423	19.6	21.9	74.85	74.92	15.53	
40–49	2,637	21.4	22.8	74.40	74.34	15.48	
50–59	2,183	17.7	17.4	72.48	72.86	15.30	
60–69	2,010	16.3	12.2	68.74	68.92	15.08	
≥ 70	1,550	12.6	9.2	62.23	61.99	15.98	
Residence							<0.0001
Urban	5,611	45.4	47.6	72.83	73.58	16.05	
Rural	6,739	54.6	52.4	71.29	72.11	16.32	
Marital status							<0.0001
Married	8,975	72.7	70.3	72.26	72.84	15.63	
Widowed/divorced/unmarried	3,375	27.3	29.7	71.28	72.74	17.68	
Employment status							<0.0001
Employed	7,392	59.9	61.2	73.47	74.07	15.36	
Unemployed	4,958	40.2	38.8	69.79	70.82	17.23	
Depressive symptoms							<0.0001
Yes	1,330	10.77	10.6	61.77	62.99	18.84	
No	11,020	89.23	89.4	73.22	73.98	15.45	
Alcohol consumption							<0.0001
Never	2,551	20.7	19.1	68.52	69.58	16.94	
1 time per month	4,367	35.4	35.1	71.56	72.22	16.15	
2–3 times per week	1,837	14.9	15.8	74.40	75.12	16.06	
≥ 4 times per week	3,595	29.1	30.1	73.75	74.34	15.44	
Smoking status							<0.0001
Never smoked	7,776	63.0	63.0	71.37	72.16	16.50	
Former smoker	1,838	14.9	13.8	72.84	73.85	15.47	
Current smoker	2,736	22.2	23.2	73.18	73.96	15.78	
Exercise							<0.0001
Never	6,946	56.2	55.3	70.08	71.08	16.84	
1–2 times per week	1,454	11.8	12.8	75.64	75.97	14.58	
3–4 times per week	1,438	11.6	12.0	74.45	74.92	15.29	
5–6 times per week	1,404	11.4	11.8	75.19	75.49	15.07	
Every day	1,108	9.0	8.2	71.89	72.61	15.30	
Chronic disease							<.0001
Yes	6,896	55.8	50.9	68.73	69.43	16.50	
No	5,454	44.2	49.1	76.11	76.31	14.96	
Total	12,350	100.0	100.0	71.99	72.81	16.21	

*EQ-VAS* EuroQol-visual analogue scale

Table 2 shows the number of participants and mean health-related quality of life at baseline according to gap classification. The analysis of mean health-related quality of life according to the income, education, and subjective social class levels revealed a significant positive correlation between the weighted mean health-related quality of life and income (Low, 68.15; Medium, 73.95; High, 76.03; *p* < 0.0001), education (Middle school or lower, 66.56; High school, 73.94; College or higher, 76.43; *p* < 0.0001), and subjective social class (Low, 69.05; Medium, 73.50; High, 75.85; *p* < 0.0001). Not all participants in the high income and education brackets rated their subjective class as high, and, similarly, not all participants with a low SES rated their subjective class as low.

**Table 2:** Health-related quality of life according to variables of interest at baseline (2009)

	Total	Unweighted %	Weighted %	Health-related quality of life (EQ-VAS)			*p* value
				Unweighted Mean	Weighted Mean	SD	
Gap between income and subjective social class							<0.0001
HH	2,263	18.3	20.2	76.77	77.00	14.28	
HM	838	6.8	7.5	74.78	75.30	15.11	
HL	645	5.2	5.7	73.09	73.56	17.46	
MH	1,493	12.1	12.6	75.39	75.57	14.99	
MM	1,061	8.6	9.0	73.85	74.06	14.63	
ML	1,605	13.0	13.3	71.74	72.34	16.51	
LH	1,069	8.7	7.6	72.84	73.26	15.08	
LM	854	6.9	6.3	69.79	70.52	15.45	
LL	2,522	20.4	17.7	64.24	65.12	17.24	
Gap between education and subjective social class							<0.0001
HH	2,276	18.4	20.8	77.08	77.22	14.65	
HM	954	7.7	9.0	76.18	76.44	14.99	
HL	870	7.0	8.2	74.03	74.44	16.93	
MH	1,562	12.7	13.0	76.11	76.15	14.22	
MM	987	8.0	8.3	73.38	73.41	14.49	
ML	1,485	12.0	12.6	71.66	71.99	17.02	
LH	987	8.0	6.7	70.77	70.99	14.96	
LM	812	6.6	5.5	68.37	68.79	15.12	
LL	2,417	19.6	15.9	63.50	63.93	16.71	
Income							<0.0001
Low	4,445	36.0	31.7	67.38	68.15	16.74	
Medium	4,159	33.7	34.9	73.59	73.95	15.57	
High	3,746	30.3	33.4	75.69	76.03	15.12	
Subjective social class							<0.0001
Low	4,772	38.6	36.8	67.96	69.05	17.44	
Medium	2,753	22.3	22.8	72.87	73.50	15.15	
High	4,825	39.1	40.5	75.47	75.85	14.75	
Education							<0.0001
≤ Middle school	4,216	34.1	28.1	66.14	66.56	16.26	
High school	4,034	32.7	33.9	73.80	73.94	15.49	
≥ College	4,100	33.2	38.0	76.22	76.43	15.28	
Total	12,350	100.0	100.0	71.99	72.81	16.21	

*EQ-VAS* EuroQol-visual analogue scale, *HH* High–High, *HM* High–Medium, *HL* High–Low, *MH* Medium–High, *MM* Medium–Medium, *ML* Medium–Low, *LH* Low–High, *LM* Low–Medium, *LL* Low–Low

**Table 3:** Adjusted effect of objective and subjective social class on health-realted quality of life

	Quality of life				
	Estimate	SE	95 % CI		*P*-value
Income					
Low	−3.3404	0.2728	−3.875	−2.806	<.0001
Middle	−1.4308	0.2253	−1.872	−0.989	<.0001
High	ref				
Education					
Low	−4.4015	0.3285	−5.045	−3.758	<.0001
Middle	−1.1785	0.2378	−1.645	−0.712	<.0001
High	ref				
Subjective social class					
Low	−4.7276	0.2228	−5.164	−4.291	<.0001
Middle	−1.8744	0.2450	−2.355	−1.394	<.0001
High	ref				

Adjusted for gender, age, residential region, marital status, economic activity status, depressive symptoms, alcohol consumption, smoking status, exercise, chronic disease, and year

We found that when subjective social status was rated one level below objective social status (i.e., High, Medium, and Low income and education levels), average health-related quality of life decreased for income (HH, HM, and HL: 77.00, 75.30, and 73.56, respectively; MH, MM, and ML: 75.57, 74.06, and 72.34, respectively; and LH, LM, and LL: 73.26, 70.52, and 65.12 respectively; *p* < 0.0001) and education (HH, HM, and HL: 77.22, 76.44, and 74.44, respectively; MH, MM, and ML: 76.15, 73.41, and 71.99, respectively; and LH, LM, and LL: 70.99, 68.79, and 63.93, respectively; *p* < 0.0001).

**Fig. 1:**
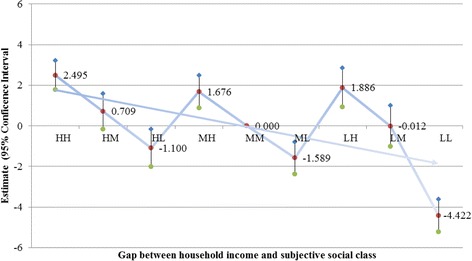
Adjusted effect of gap between income and subjective social class on health-related quality of life. HH, High–High; HM, High–Medium; HL, High–Low; MH, Medium–High; MM, Medium–Medium; ML, Medium–Low; LH, Low–High; LM, Low–Medium; LL, Low–Low

**Fig. 2:**
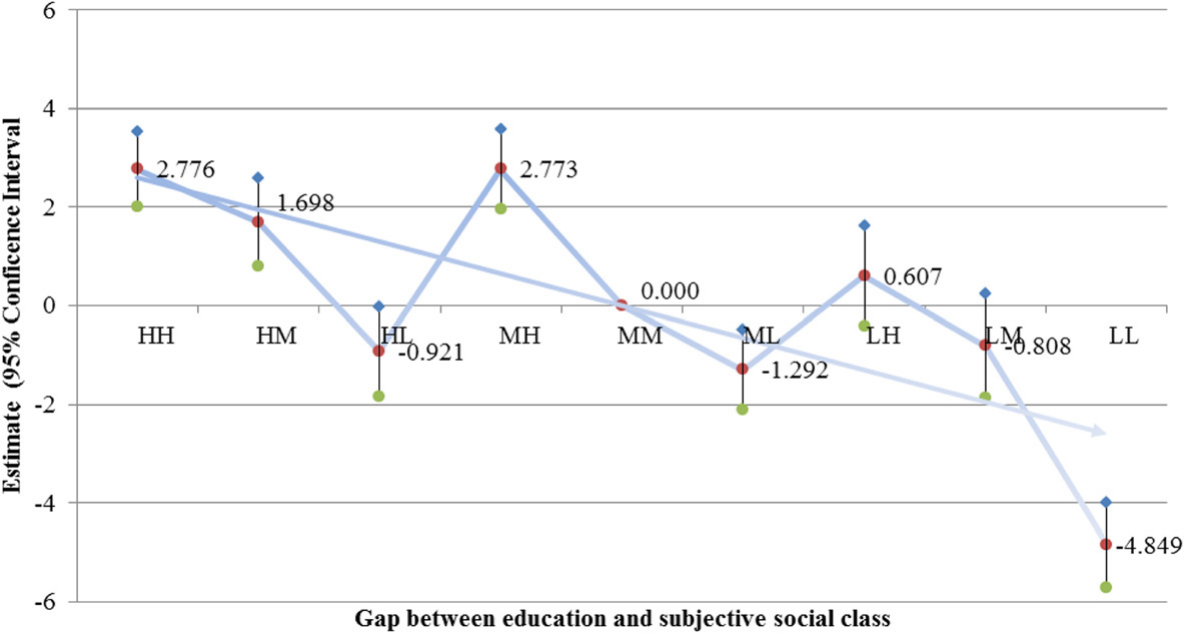
Adjusted effect of gap between education and subjective social class on health-related quality of life. HH, High–High; HM, High–Medium; HL, High–Low; MH, Medium–High; MM, Medium–Medium; ML, Medium–Low; LH, Low–High; LM, Low–Medium; LL, Low–Low

We analyzed the relationship between each social status (income, education, and subjective social class) and health-related quality of life controlling for all covariates to investigate whether low social status was associated with poor health-related quality of life (see Table 3). Table 4, Figs. 1 and 2 show the results of the linear mixed model analysis that assessed the effect of the gap between objective and subjective social status on health-related quality of life. After controlling for the influence of covariates, the HH group had the highest perceived health state (2.495, 2.776 [*p* < 0.0001] for income and education), while the LL group had the lowest perceived health state (−4.422, −4.849 [*p* < 0.0001] for income and education). In the objective social class dimension, we found that the difference in estimation was as follows: when household income was high, the high to low subjective social status was 2.495 (*p* < 0.0001), 0.709 (*p* = 0.112), and −1.110 (*p* = 0.020), respectively. For mid-level income the high and low subjective social status was 1.676 and −1.589, respectively (*p* < 0.0001 for both); and for low income the high to low subjective social status was 1.886 (*p* < 0.0001), −0.012 (*p* = 0.982), and −4.422 (*p* < 0.0001) using the MM group as the reference. The results were similar for education: for the high-income level the high to low subjective social status was 2.776 (*p* < 0.0001), 1.698 (*p =* 0.000), and −0.921 (*p* < 0.047), respectively; for mid-level education the high and low subjective social status was 2.773 (*p* < 0.0001) and −1.292 (*p* = 0.002), respectively; and for the low-education level the high to low subjective social status was 0.607 (*p* = 0.245), −0.808 (*p* = 0.135), and −4.849 (*p* < 0.0001) using the MM group as the reference. There are almost similar trends in both male and female (Table S1, S2, Figure S1, S2, S3, and S4).

**Table 4:** Effect of objective and subjective social status on health-related quality of life

	Household income					Education				
	Estimate	SE	95 % CI		P-value	Estimate	SE	95 % CI		*P*-value
Gap between Income and Subjective Social Class										
HH	2.495	0.369	1.772	3.218	<.0001	2.776	0.390	2.011	3.540	<.0001
HM	0.709	0.446	−0.165	1.583	0.112	1.698	0.456	0.805	2.591	0.000
HL	−1.100	0.472	−2.025	−0.175	0.020	−0.921	0.463	−1.828	−0.014	0.047
MH	1.676	0.411	0.870	2.482	<.0001	2.773	0.417	1.955	3.590	<.0001
MM	ref					ref				
ML	−1.589	0.400	−2.373	−0.804	<.0001	−1.292	0.418	−2.111	−0.473	0.002
LH	1.886	0.495	0.916	2.856	0.000	0.607	0.521	−0.415	1.629	0.245
LM	−0.012	0.517	−1.025	1.001	0.982	−0.808	0.540	−1.867	0.251	0.135
LL	−4.422	0.408	−5.221	−3.623	<.0001	−4.849	0.441	−5.714	−3.984	<.0001
Gender										
Male	ref					ref				
Female	−2.572	0.289	−3.139	−2.005	<.0001	−2.213	0.292	−2.785	−1.640	<.0001
Age										
≤ 29	7.960	0.471	7.037	8.884	<.0001	6.631	0.507	5.638	7.624	<.0001
30-39	6.692	0.428	5.853	7.531	<.0001	5.416	0.461	4.513	6.319	<.0001
40-49	6.586	0.419	5.764	7.407	<.0001	5.586	0.440	4.724	6.449	<.0001
50-59	5.742	0.414	4.932	6.553	<.0001	5.602	0.410	4.799	6.405	<.0001
60-69	4.284	0.415	3.470	5.098	<.0001	4.310	0.414	3.497	5.122	<.0001
≥ 70	1.000					1.000				
Residential region										
Urban	ref					ref				
Rural	−0.565	0.190	−0.937	−0.193	0.003	−0.536	0.190	−0.908	−0.164	0.005
Marital status										
Married	ref					ref				
Single	−0.016	0.256	−0.519	0.486	0.949	−0.036	0.256	−0.538	0.467	0.890
Economic activity status										
Yes	ref					ref				
No	−0.556	0.216	−0.979	−0.133	0.010	−0.899	0.214	−1.319	−0.480	<.0001
Depressive symptom										
Yes	−9.607	0.329	−10.252	−8.963	<.0001	−9.651	0.328	−10.295	−9.007	<.0001
No	ref					ref				
Alcohol consumption										
Never	1.000					1.000				
1 times per month	0.311	0.270	−0.218	0.839	0.249	0.344	0.270	−0.185	0.872	0.202
2-3 times per week	0.552	0.341	−0.116	1.219	0.105	0.630	0.340	−0.036	1.297	0.064
≥ 1 times per week	0.018	0.304	−0.577	0.613	0.953	0.122	0.303	−0.472	0.717	0.687
Smoking status										
Never smoker	ref					ref				
Former smoker	0.465	0.356	−0.234	1.163	0.193	0.329	0.357	−0.370	1.028	0.356
Current smoker	−1.547	0.321	−2.175	−0.918	<.0001	−1.562	0.321	−2.190	−0.933	<.0001
Exercise										
Never	ref					ref				
1-2 times per week	1.691	0.289	1.125	2.257	<.0001	1.605	0.290	1.038	2.172	<.0001
3-4 times per week	2.497	0.298	1.913	3.081	<.0001	2.444	0.298	1.861	3.028	<.0001
5-6 times per week	2.839	0.318	2.216	3.462	<.0001	2.889	0.318	2.266	3.511	<.0001
Everyday	1.700	0.395	0.927	2.474	<.0001	1.758	0.395	0.985	2.532	<.0001
Chronic disease										
Yes	−3.304	0.220	−3.736	−2.873	<.0001	−3.235	0.221	−3.668	−2.803	<.0001
No	ref					ref				
Year										
2009	1.657	0.190	1.285	2.029	<.0001	1.607	0.189	1.237	1.977	<.0001
2011	ref					ref				

*HH* High–High, *HM* High–Medium, *HL* High–Low, *MH* Medium–High, *MM* Medium–Medium, *ML* Medium–Low, *LH* Low–High, *LM* Low–Medium, *LL* Low–Low

## Discussion

We investigated the impact of a gap between objective and subjective social class on health-related quality of life. Our finding of an association between social status (income, education, and subjective social class) and health-related quality of life was consistent with previous studies [17, 19, 49, 50]. We extended this finding by investigating the impact of the disparity between objective and subjective social status on health-related quality of life. The results revealed that the higher status is related to higher health-related quality of life, whereas the lower status is related to lower health-related quality of life. At any given objective social class (i.e., High, Medium, and Low), an individual’s health-related quality of life declined with a one-level decrease in subjective social status. Education also showed a similar trend.

One of our aims was to determine whether social status, as defined by income, education, and subjective social position, was associated with health-related quality of life. Our results revealed that health-related quality of life was significantly worse in respondents with lower incomes, education levels, and perceived social status compared to those with higher SES and subjective social class ratings. These results support previous findings of an association between objective social class and health-related quality of life or self-rated health [19, 49, 50]. A study found a strong association between annual household income and health-related quality of life as measured using the SF-36 [50]. Another study found that SF-36 scores increased with better housing type and higher education level after the authors adjusted for the influence of the determinants of health-related quality of life in an urban Asian population [49].

The association between subjective social class and health-related quality of life (or self-rated health) has been investigated in a number of countries [14, 15, 17, 21, 22, 35, 36]. Goodman et al. reported that lower subjective social status and changes in subjective social status predicted poor self-rated health [21]. Moreover, a study of elderly people in Taiwan found that lower subjective social status predicted decline in health beyond that accounted for by objective indicators of SES. The effect was significantly reduced in all health outcomes compared to in controls [14]. Another study reported that after household income was adjusted, subjective social status was significantly associated with self-rated health among White and Chinese American pregnant women [36].

Our main results reveal that gaps between objective and subjective social status were significantly associated with health-related quality life. Although previous studies have shown that subjective social status, typically measured by asking respondents to assess their social status relative to others, was correlated with objective social strata, including income and education [10, 11, 36, 37], such studies have not investigated the perceived social status of respondents with high objective social status. To further explore the impact of the disparity between objective and perceived social status, we placed respondents into nine gap categories according to the difference between their objective and subjective social class. We found that health-related quality of life decreased as subjective social class decreased in individuals who reported a high (middle or low) objective social class (Figs. 1 and 2).

Based on existing literature, two psychosocial mechanisms may explain the gap between objective and subjective social class on health outcome. First, people with lower subjective social status are more likely to perceive economic strain, insufficiency, and financial insecurity regarding the future [17]. These unfavorable perceptions may increase anxiety and the sense of vulnerability, leading to adverse health consequences [10]. Second, we may explain why the gap between objective and subjective social class may occur through the reference group theory and how the gap may affect health. People, by comparing themselves with others, feel that their socioeconomic status is insufficient for participation in the lifestyles or norms (e.g., healthy lifestyles or behaviors) of their peer group; and consequently, their health is affected [51]. Moreover, a study indicated that a person’s perception of his or her SES may play a crucial role in mediating the relationship between objective class (e.g., education or occupation) and various health outcomes [17]. Indeed, our results show that not all participants with high incomes and educational attainment reported a high health-related quality of life, just as not all participants with low incomes and education levels reported a poor health-related quality of life. In other words, individuals who rated themselves as having a high social status did not always perceive their health state as high. These findings indicate that further evidence for the importance of subjective social status for health-related quality of life.

Our study has several limitations. First, although the instrument we used to measure health-related quality of life is widely accepted as a valid and reliable scale [45], the subjective nature of the questionnaire may have introduced bias into the study. Future studies should use objective health outcome variables (i.e., clinical measurements of health) rather than a single questionnaire to assess health-related quality of life. Second, our analysis was limited to two time points owing to the absence of data. Future studies should investigate over a longer time period. Despite these limitations, our study has novel implication. Our results are generalizable to the wider Korean population because we used data from a large, nationally representative, longitudinal survey.

## Conclusion

Several previous studies have established the relationship between the effects of SES on health outcomes and health-related quality of life. However, we found that the higher social class is related to the higher health-related quality of life and health-related quality of life varies according to perceived social status at the same level of objective social class. Thus, socioeconomic inequalities should be taken into account using multidimensional measurement tools rather than a single measure when designing health interventions.

## Additional file

Additional file 1:
**Table S1.** Adjusted effect of income and subjective social status on health-related quality of life according to sex. **Table S2.** Adjusted effect of education and subjective social status on health-related quality of life according to sex. **Figure S1.** Adjusted effect of income and subjective social status on health-related quality of life in male. **Figure S2.** Adjusted effect of income and subjective social status on health-related quality of life in female. **Figure S3.** Adjusted effect of education and subjective social status on health-related quality of life in male. **Figure S4.** Adjusted effect of education and subjective social status on health-related quality of life in female. (DOC 254 kb)

## Abbreviations

AIMS: The Arthritis Impact Measurement Scale
EQ-VAS: The Euroqol visual analogue scale
EQ-5D: The five dimensions of the EuroQoL questionnaire
FACT-L: The Functional Assessment of Cancer Therapy-Lung
HRQoL: Health-related quality of life
NHP: The Nottingham Health Profile
SCI-QL 23: The Spinal Cord Injury Quality of Life Questionnaire 23
SES: Socioeconomic status
SF-36: The Short-Form Health Survey
