# Supplementary material for: The effect of subjective and objective social class on health-related quality of life: new paradigm using longitudinal analysis
Source: Health Qual Life Outcomes. 2015 Aug 8;13:121. doi: 10.1186/s12955-015-0319-0 (PMC4529728; doi:10.1186/s12955-015-0319-0)

Supplementary table 2. Adjusted effect of income and subjective social status on health-related quality of life according to sex

|  | **Household income (Male)** | | | | |  | **Household income (Female)** | | | | |
| --- | --- | --- | --- | --- | --- | --- | --- | --- | --- | --- | --- |
|  | **Estimate** | **SE** | **95% CI** | | **P-value** |  | **Estimate** | **SE** | **95% CI** | | **P-value** |
| **Gap between Income and Subjective Social Class** |  |  |  |  |  |  |  |  |  |  |  |
| **HH** | 2.354 | 0.529 | 1.317 | 3.392 | <.0001 |  | 2.638 | 0.511 | 1.636 | 3.640 | <.0001 |
| **HM** | 0.783 | 0.636 | -0.464 | 2.030 | 0.219 |  | 0.640 | 0.621 | -0.576 | 1.856 | 0.302 |
| **HL** | -1.459 | 0.664 | -2.761 | -0.157 | 0.028 |  | -0.779 | 0.666 | -2.085 | 0.528 | 0.243 |
| **MH** | 1.725 | 0.592 | 0.563 | 2.886 | 0.004 |  | 1.624 | 0.568 | 0.510 | 2.738 | 0.004 |
| **MM** | ref |  |  |  |  |  | ref |  |  |  |  |
| **ML** | -1.367 | 0.568 | -2.480 | -0.254 | 0.016 |  | -1.788 | 0.561 | -2.888 | -0.689 | 0.001 |
| **LH** | 1.961 | 0.741 | 0.510 | 3.412 | 0.008 |  | 1.869 | 0.667 | 0.562 | 3.176 | 0.005 |
| **LM** | -1.164 | 0.762 | -2.658 | 0.329 | 0.127 |  | 0.885 | 0.702 | -0.491 | 2.261 | 0.208 |
| **LL** | -4.962 | 0.598 | -6.134 | -3.789 | <.0001 |  | -3.899 | 0.558 | -4.992 | -2.806 | <.0001 |
| Adjusted for age, residential region marital status, economic activity status, depressive symptom, alcohol consumption, smoking status, exercise, chronic disease, and year  EQ-VAS, EuroQol-visual analogue scale; HH, High–High; HM, High–Medium; HL, High–Low; MH, Medium–High; MM, Medium–Medium; ML, Medium–Low; LH, Low–High; LM, Low–Medium; LL, Low–Low. | | | | | | | | | | | |

Supplementary table 3. Adjusted effect of education and subjective social status on health-related quality of life according to sex

|  | **Education (Male)** | | | | |  | **Education (Female)** | | | | |
| --- | --- | --- | --- | --- | --- | --- | --- | --- | --- | --- | --- |
|  | **Estimate** | **SE** | **95% CI** | | **P-value** |  | **Estimate** | **SE** | **95% CI** | | **P-value** |
| **Gap between Income and Subjective Social Class** |  |  |  |  |  |  |  |  |  |  |  |
| **HH** | 3.105 | 0.550 | 2.026 | 4.183 | <.0001 |  | 2.681 | 0.552 | 1.599 | 3.764 | <.0001 |
| **HM** | 2.253 | 0.634 | 1.010 | 3.497 | 0.000 |  | 1.375 | 0.653 | 0.095 | 2.655 | 0.035 |
| **HL** | -0.981 | 0.631 | -2.217 | 0.255 | 0.120 |  | -0.411 | 0.682 | -1.747 | 0.925 | 0.547 |
| **MH** | 3.057 | 0.613 | 1.855 | 4.260 | <.0001 |  | 2.483 | 0.568 | 1.369 | 3.596 | <.0001 |
| **MM** | ref |  |  |  |  |  | ref |  |  |  |  |
| **ML** | -1.213 | 0.590 | -2.370 | -0.057 | 0.040 |  | -1.398 | 0.588 | -2.551 | -0.244 | 0.018 |
| **LH** | 1.401 | 0.823 | -0.212 | 3.015 | 0.089 |  | 0.251 | 0.691 | -1.103 | 1.604 | 0.717 |
| **LM** | -1.203 | 0.841 | -2.852 | 0.446 | 0.153 |  | -0.661 | 0.718 | -2.068 | 0.745 | 0.357 |
| **LL** | -4.076 | 0.644 | -5.338 | -2.813 | <.0001 |  | -5.188 | 0.612 | -6.388 | -3.989 | <.0001 |
| Adjusted for age, residential region marital status, economic activity status, depressive symptom, alcohol consumption, smoking status, exercise, chronic disease, and year  HH, High–High; HM, High–Medium; HL, High–Low; MH, Medium–High; MM, Medium–Medium; ML, Medium–Low; LH, Low–High; LM, Low–Medium; LL, Low–Low. | | | | | | | | | | | |

Supplementary figure 1. Adjusted effect of income and subjective social status on health-related quality of life


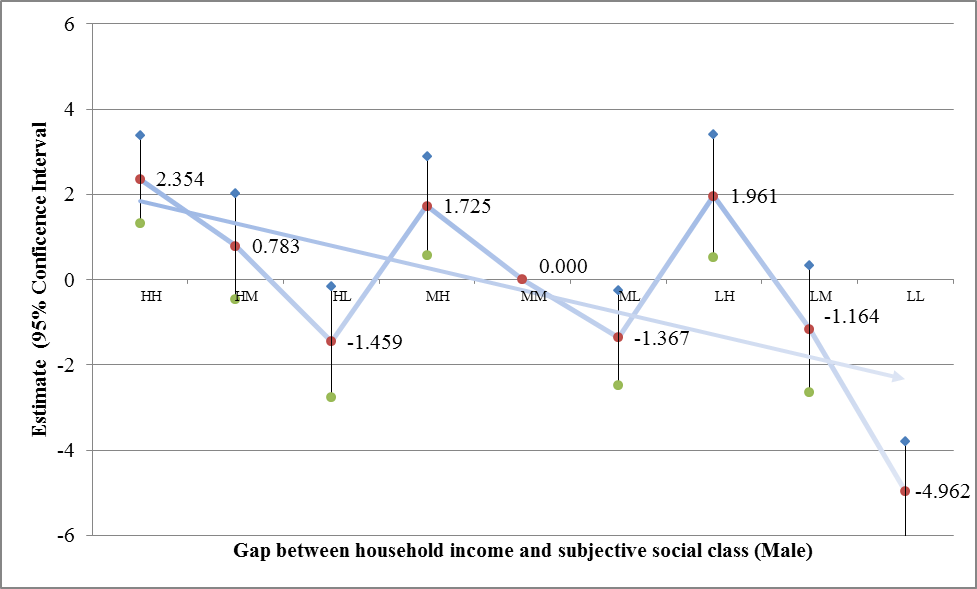


HH, High–High; HM, High–Medium; HL, High–Low; MH, Medium–High; MM, Medium–Medium; ML, Medium–Low; LH, Low–High; LM, Low–Medium; LL, Low–Low.

Supplementary figure 2. Adjusted effect of income and subjective social status on health-related quality of life


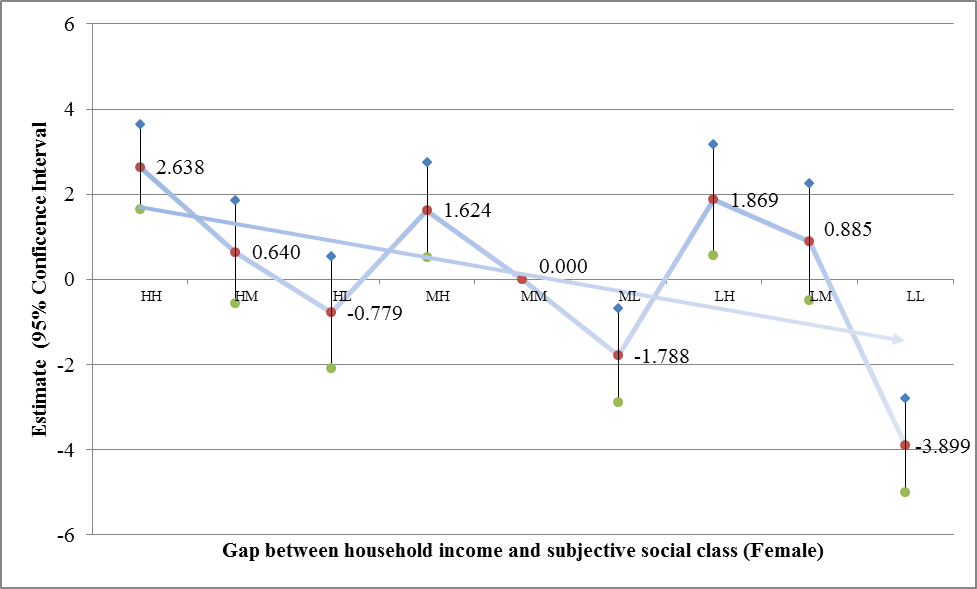


HH, High–High; HM, High–Medium; HL, High–Low; MH, Medium–High; MM, Medium–Medium; ML, Medium–Low; LH, Low–High; LM, Low–Medium; LL, Low–Low.

Supplementary figure 3. Adjusted effect of education and subjective social status on health-related quality of life


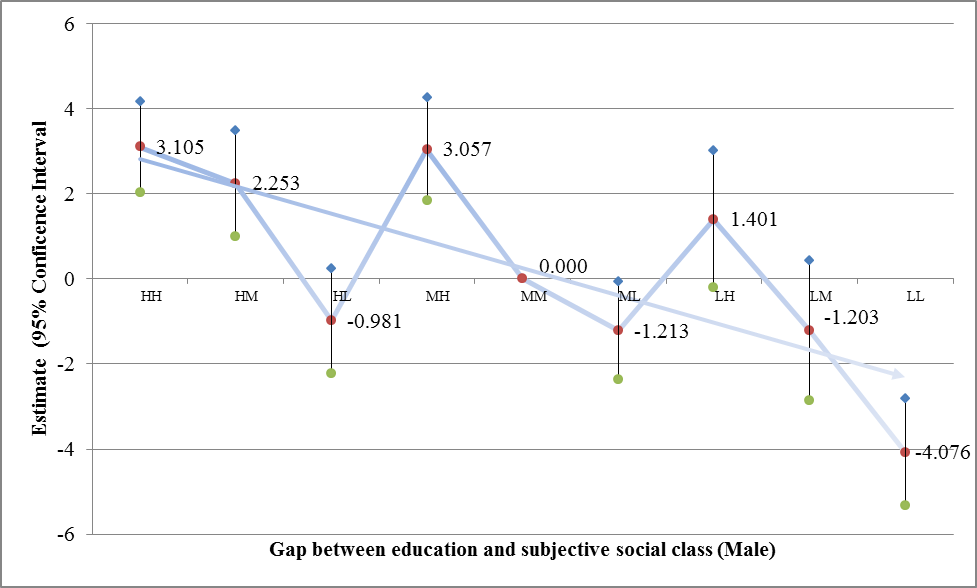


Supplementary figure 4 Adjusted effect of education and subjective social status on health-related quality of life


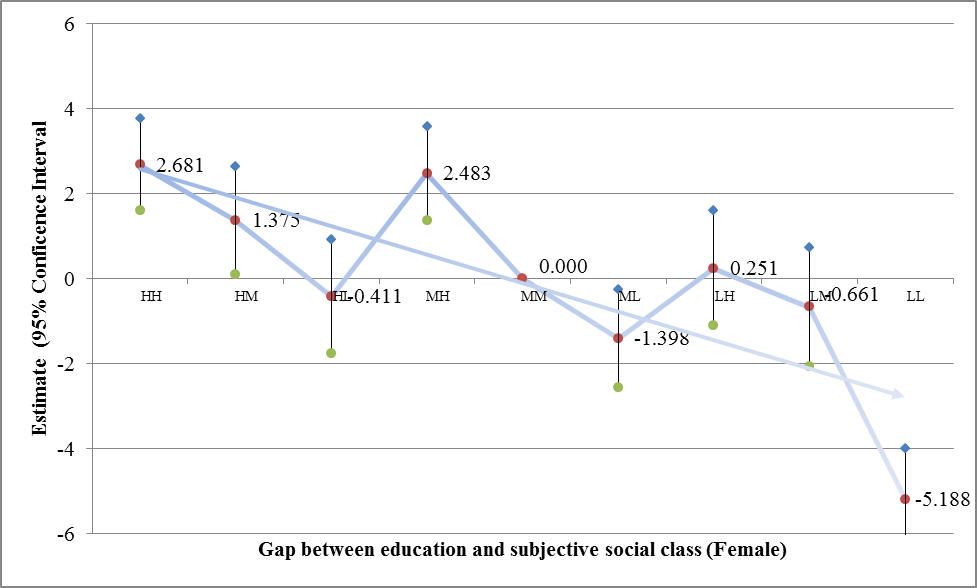

Supplement: Additional file 1: — Table S1. Adjusted effect of income and subjective social status on health-related quality of life according to sex. Table S2. Adjusted effect of education and subjective social status on health-related quality of life according to sex. Figure S1. Adjusted effect of income and subjective social status on health-related quality of life in male. Figure S2. Adjusted effect of income and subjective social status on health-related quality of life in female. Figure S3. Adjusted effect of education and subjective social status on health-related quality of life in male. Figure S4. Adjusted effect of education and subjective social status on health-related quality of life in female. (DOC 254 kb) [file 12955_2015_319_MOESM1_ESM.doc]
